# Stability of alglucosidase alfa in 0.9% sodium chloride for enzyme replacement therapy in patients with Pompe disease: insights from enzyme activity and cellular uptake measurements

**DOI:** 10.1136/ejhpharm-2025-004516

**Published:** 2025-08-17

**Authors:** Ina Barzel, Jan-Dietert C Brugma, Edwin H Jacobs, Marianne Hoogeveen-Westerveld, P Hugo M van der Kuy, Tim Preijers

**Affiliations:** 1 Department of Hospital Pharmacy, Erasmus University Medical Center, Rotterdam, The Netherlands; 2 Department of Outpatient Pharmacy, Erasmus University Medical Center, Rotterdam, The Netherlands; 3 Department of Clinical Genetics, Erasmus University Medical Center, Rotterdam, The Netherlands

**Keywords:** Drug Stability, Drug Storage, CLINICAL MEDICINE, DRUG STABILITY, Analytic Sample Preparation Methods, Chemistry, Pharmaceutical, NEUROLOGY, Administration, Intravenous, PHARMACY SERVICE, HOSPITAL

## Abstract

**Objectives:**

Enzyme replacement therapy (ERT) with alglucosidase alfa is the cornerstone of treatment for Pompe disease, a rare disorder caused by acid α-glucosidase (GAA) deficiency. Since 2008, home infusions have been provided in the Netherlands. However, the short shelf-life of the ready-to-administer infusions poses challenges for manufacturing and dispensing pharmacies. This study assessed the stability of ready-to-administer alglucosidase alfa infusions by determining enzyme activity and cellular uptake of two concentrations during 11 days of storage.

**Methods:**

Alglucosidase alfa infusions (2 and 4 mg/mL in 0.9% sodium chloride) were prepared and samples were drawn on days 1 to 7 and 11. Enzyme activity was determined using 4-methylumbelliferyl-α-D-glucoside (4MU-αGlc) and glycogen as substrates. Additionally, enzyme uptake in cultured fibroblasts was investigated.

**Results:**

There was no difference in enzyme activity after 11 days as compared with day 1 using 4MU-αGlc (2 mg/mL: 352 vs 331 nmol/h/mL; 4 mg/mL: 657 vs 662 nmol/h/mL) or glycogen (2 mg/mL: 183 vs 176 nmol/h/mL; 4 mg/mL: 352 vs 357 nmol/h/mL). Uptake of alglucosidase alfa in fibroblasts remained stable over 11 days, with activity ranging from 90 to 104 nmol/h/mL at 2 mg/mL and from 233 to 238 nmol/h/mL at 4 mg/mL. Linear regression analysis confirmed no statistically significant association between time and enzyme activity or uptake.

**Conclusions:**

Ready-to-administer alglucosidase alfa infusion in 0.9% sodium chloride at 2–4 mg/mL is stable for 11 days when stored at 2–8°C or −20°C and protected from light. Extending the stability could enhance efficiency and flexibility for infusion preparation and home delivery, while minimising pharmaceutical waste.

**WHAT IS ALREADY KNOWN ON THIS TOPIC**

- Home infusion therapy is rapidly expanding due to its cost-efficiency and patient convenience. However, the limited 24-hour stability of centrally prepared, costly alglucosidase alfa infusions for Pompe disease challenges its adoption.

**WHAT THIS STUDY ADDS**

- The enzymatic activity of alglucosidase alfa infusions remains stable for 11 days at concentrations between 2 and 4 mg/mL.

**HOW THIS STUDY MIGHT AFFECT RESEARCH, PRACTICE OR POLICY**

- This study supports the global implementation of home infusion therapy with alglucosidase alfa for patients with Pompe disease.

## Introduction

 Since 2006, enzyme replacement therapy (ERT) with alglucosidase alfa (Myozyme®) has been the standard of care for patients with Pompe disease, a rare, inheritable and progressive lysosomal storage disorder caused by a deficiency of the enzyme acid α-glucosidase (GAA). Deficient enzymatic activity leads to the accumulation of lysosomal glycogen in various tissues, primarily causing progressive muscle damage resulting in loss of respiratory function.^1 2^ Alglucosidase alfa is a recombinant human acid α-glucosidase (rhGAA) and has been licensed for the treatment of Pompe disease based on its ability to improve walking distance, stabilise pulmonary function and improve survival; it is administered intravenously at a dosage of 20 mg/kg bi-weekly.^2–12^ While ERT has positively altered the disease course for most patients, their quality of life is negatively impacted by the time-consuming infusions taking several hours.^13^

All patients with Pompe disease in the Netherlands receive treatment at the Center for Lysosomal and Metabolic Diseases of the Erasmus University Medical Center (Erasmus MC) in Rotterdam, which is the national referral centre for Pompe disease. In 2008, the Netherlands became the first country to implement home-based infusions of alglucosidase alfa. Patients with Pompe disease in the Netherlands are eligible for home infusions after 1 year of uncomplicated treatment in the hospital.^14^ Currently, around 80% of these patients receive infusions at home, as it increases patient flexibility and autonomy, avoids travel time of often several hours and offers a more patient-centred approach to ERT.^15^ According to the manufacturer of Myozyme®, infusions are chemically and physically stable for 24 hours between 2–8°C after preparation (ready-to-administer) when stored under protection from light.^12^ However, this poses logistical challenges, as the infusions are centrally prepared in the hospital pharmacy of the Erasmus MC and have to be transported to patients throughout the Netherlands. In addition, there is a constant risk of therapy being postponed at short notice after infusion preparation, leading to potential medication waste of this high-cost drug. In 2020, expenses for alglucosidase alfa for the treatment of 136 patients in the Netherlands amounted to approximately €69 million.^16^

The GAA enzymatic activity assay serves as a critical tool in Pompe disease diagnosis and research, particularly in evaluating the efficacy of ERT.^1 12^ ERT is focused on delivering recombinant lysosomal enzymes, such as alglucosidase alfa, to lysosomes within cells. This delivery process relies on cation-independent mannose-6-phosphate receptors (CI-MPRs) present on the surface of, among others, cardiomyocytes and skeletal muscle cells, the primary sites affected in Pompe disease.^17–19^ Within lysosomes, alglucosidase alfa performs its enzymatic function by breaking down glycogen into glucose. As an increased storage duration may affect the physical and chemical stability of rhGAA, the evaluation of the enzyme uptake and activity under altered storage duration and condition is essential, ensuring structural and functional integrity of rhGAA over time.

With a focus on enabling home treatment for all patients with Pompe disease, this study aimed to evaluate the functional stability of two concentrations of ready-to-administer alglucosidase alfa infusions by determining the enzyme uptake and activity in cultured human skin fibroblasts over a period of 11 days.

## Materials and methods

An experimental study was performed in the hospital pharmacy of the Erasmus University Medical Center in Rotterdam, the Netherlands. This study was performed under the required conditions for preparing medication in the hospital. As this study did not involve human or animal subjects, ethical approval was not necessary.

### Preparation of infusion bags

For preparation of the infusions, the following materials were used: needles (19G×1½’’, 1.1 mm × 40 mm, Becton, Dickinson and Company (BD)), syringes (1, 3 and 10 mL, BD) and infusion bags (EVA 150 mL, Baxter International Inc.). Preparation of the infusions and sample collection took place in laminar air flow (LAF) cabinets in the cleanrooms of the hospital pharmacy. On day 0, two vials of lyophilised alglucosidase alfa (50 mg per vial) were each reconstituted with 10.3 mL water for injections, achieving a final concentration of 5 mg/mL. From each vial, a 1 mL sample was taken and frozen. The remaining solution of 9 mL (45 mg alglucosidase alfa) was added to 13.5 mL and 2.25 mL of 0.9% sodium chloride to obtain a final concentration of 2 mg/mL and 4 mg/mL, respectively.

### Sample collection

On days 1 to 7 and on day 11, 1 mL of solution was drawn from each of the 2 and 4 mg/mL infusion bags. The samples were preserved in light-proof bags at −20°C in a temperature-controlled freezer, while the infusion bags were stored in a light-proof bag at 2–8°C in a temperature-controlled fridge.

### Determination of enzyme activity

To determine the alglucosidase alfa activity of the prepared infusions (2 and 4 mg/mL), two assays were employed, which are routinely used in our laboratory to determine GAA activity in leukocytes. These assays differed in the substrate used: either the natural substrate glycogen or the artificial substrate 4-methylumbelliferyl-α-D-glucopyranoside (4MU-αGlc).^20^

Ten microlitres of 6000 or 12 000 times diluted sample, 20 µL substrate (7.5% w/v glycogen (from rabbit liver; Sigma type III) and 4.5 µmol/L acarbose (Toronto Research Chemicals, North York, ON, Canada) in McIlvain’s Pi/Ci buffer, pH 4.0 + 0.02% w/v sodium azide) were mixed and incubated at 37°C for 2 hours. Reactions were terminated by boiling for 2 min in a water bath. After cooling, 10 µL of 0.1 mol/L *N*-ethylmaleimide (NEM) in water, freshly prepared, was added, after which 300 µL glucose reagent was added (2.1 mmol/L 2,2‘-azino-di-[3-ethylbenzthiazolinsulfonate-6] in 0.5 mol/L Tris-HCl buffer (pH 7.0) containing 10 mmol/L magnesium chloride, 0.02% w/v sodium azide, glucose oxidase (10 U/mL; from *A. niger*, Roche grade I) and peroxidase (2.5 U/mL; from horseradish, Roche grade I)) to start colour development at room temperature for 75 min. During this incubation, the mixtures were centrifuged at room temperature for 5 min at 10 000 rounds per min and the supernatant was transferred to a 96-well plate. After 75 min, the absorbance at 420 nm was read in a plate reader (VarioSkan; Thermo Electron Corporation/Thermo Fisher Scientific Inc., Waltham, MA, USA).

For the determination of alglucosidase alfa activity with 4MU-αGlc as substrate, 10 µL of the sample (6000 or 12 000 times diluted in bovine serum albumin) was incubated with 20 µL 2.2 mM 4MU-αGlc in 0.2 M sodium acetate buffer at pH 4.0 for 1 hour at 37°C. The reactions were stopped by adding 200 µL sodium carbonate (0.5 M, pH 10.7) containing 0.25% w/v TritonX-100. The amount of fluorescence released by free 4-methylumbelliferon was determined using a plate reader (VarioSkan; Thermo Electron Corporation/Thermo Fisher Scientific Inc.) (excitation at 365 nm, emission at 448 nm) and compared with a standard concentration of 4-methylumbelliferon.

All measurements were performed in duplicate and values of the different dilutions were averaged after taking the dilution factor into account. The concentration of protein was determined by a standard bicinchoninic acid (BCA) assay.

### Determination of uptake in fibroblasts

The uptake of alglucosidase alfa was reflected by the alglucosidase alfa activity in GAA-deficient fibroblasts. This was determined by adding an amount of alglucosidase alfa equivalent to 200 nmol/h 4MU-αGlc to 200 µL medium and 20 µL fetal calf serum to the medium of the fibroblasts, 24 hours later followed by the measurement of alglucosidase alfa activity in the fibroblasts and in the medium, as described in the section ‘Determination of enzyme activity’ and by Van Gelder *et al* and De Vries *et al*.^21 22^ This experiment was performed in duplicate for each time point.

### Linear regression analysis

Linear regression modelling was performed to investigate the linear relationship between time after preparation (independent variable) and alglucosidase alfa activity (dependent variable). The estimated regression model was defined as follows:

y = α + β * x

with *y* being GAA activity, *α* the estimate of the first measured GAA activity, *β* the slope, and *x* being the time after preparation of the infusion bags.

Assumptions of linearity were verified using scatterplots. Linear modelling was performed using R version 4.2.2 (R Foundation for Statistical Computing, Vienna, Austria) and RStudio version 4.2. A p value <0.05 was considered statistically significant and a 95% confidence interval (95% CI) for the slope (*β*) was calculated.

## Results

**Figure 1:**
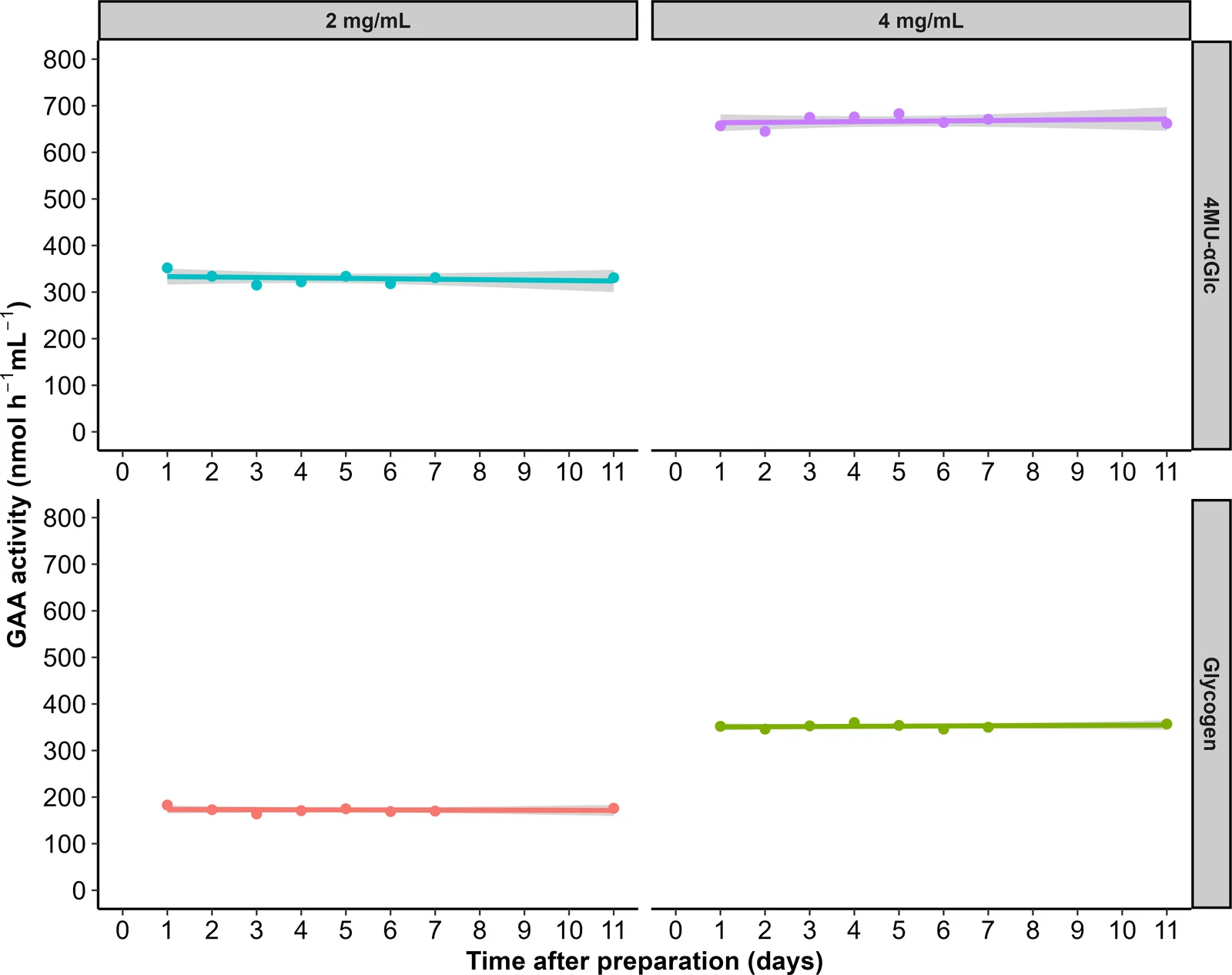
Activity of alglucosidase alfa 2 mg/L and 4 mg/L over a period of 11 days. The enzyme activity was measured using 4-methylumbelliferyl-α-D-glucopyranoside (4MU-αGlc) and glycogen as substrates. The solid line represents the regression line, the grey shaded area represents the 95% CI. GAA, acid α-glucosidase.

The alglucosidase alfa activity was determined in two ways, with 4MU-αGlc and glycogen as substrates. As shown in figure 1, there was no significant difference in alglucosidase alfa activity at both concentrations of 2 and 4 mg/mL after 11 days as compared with day 1, regardless of the substrate. With 4MU-αGlc as an artificial substrate, enzyme activity ranged from 352 to 331 nmol/h/mL at 2 mg/mL and from 657 nmol/h/mL to 662 nmol/h/mL at 4 mg/mL. Using glycogen as a natural substrate, GAA activity varied between 183 and 176 nmol/h/mL at 2 mg/mL and between 352 nmol/h/mL and 357 nmol/h/mL at 4 mg/mL.

**Figure 2:**
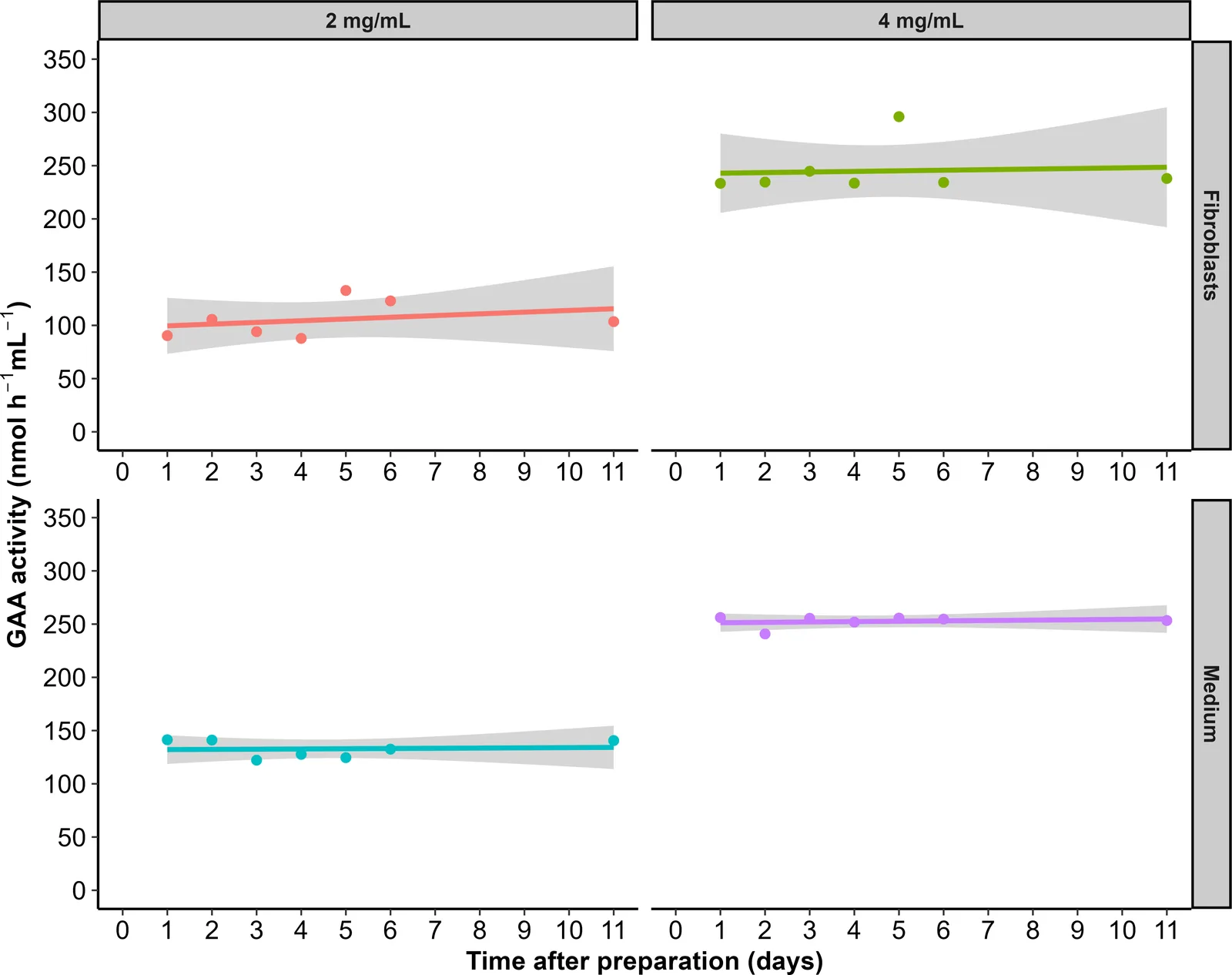
Uptake of alglucosidase alfa 2 mg/L and 4 mg/L over a period of 11 days. The enzyme activity in cultured fibroblasts and in culture medium was measured using 4-methylumbelliferyl-α-D-glucopyranoside (4MU-αGlc) as substrate. The solid line represents the regression line, the grey shaded area represents the 95% CI. GAA, acid α-glucosidase.

For the enzyme uptake analysis, comparable results were obtained as shown in figure 2. GAA activity in culture medium and in fibroblasts was used as a measure of alglucosidase alfa uptake in fibroblasts. No effect on the uptake of alglucosidase alfa was observed after 11 days as compared with day 1 at both concentrations of 2 and 4 mg/mL. The uptake of alglucosidase alfa in cultured fibroblasts remained stable, with enzyme activity in the range of 90 nmol/h/mL to 104 nmol/h/mL at 2 mg/mL and 233 nmol/h/mL to 238 nmol/h/mL at 4 mg/mL after 11 days.

In tables 1 and 2, the results from the linear regression analysis evaluating the relationship between time after preparation of the infusion bags and enzyme activity or uptake are depicted. The estimated coefficients were small (ranging from −0.94 to 1.61), indicating that alglucosidase alfa activity or uptake changed only slightly over time (day after preparation of the infusion bags). This conclusion was supported by zero being in the 95% CI of all estimated coefficients, confirming no statistically significant association between time and GAA activity or uptake. Additionally, all R squared values were close to zero (ranging from 0.01 to 0.1), suggesting that nearly none of the variance in GAA activity or uptake (<10%) was explained by time after preparation of the infusion bags.

**Table 1:** Summary statistics of the linear regression analysis of the enzyme activity

		Coefficient (*β*)				
**Concentration infusion**	**Measurement method**	**Estimate**	**95% CI**	**P value**	Linear regression model (y = α + β * x)	***R^2^***
2 mg/mL	4MU	−0.94	(−4.47 to 2.6)	0.54	*y*=334.19 + −0.94 * *x*	0.07
4 mg/mL	4MU	0.78	(−2.95 to 4.52)	0.63	*y*=662.8 + 0.78 * *x*	0.04
2 mg/mL	Glycogen	−0.17	(−1.93 to 1.58)	0.82	*y*=173.48 + −0.17 * *x*	0.01
4 mg/mL	Glycogen	0.4	(−1.09 to 1.89)	0.54	*y*=350.31 + 0.4 * *x*	0.07

R2: coefficient of determination (proportion of variance explained by the model), *y*: GAA activity, *α*: estimate of the first measured GAA activity, *β*: slope, *x*: time after preparation of the infusion bags.

A p value for *β* <0.05 is considered statistically significant.

GAA, acid α-glucosidase; 4MU, 4-methylumbelliferyl-α-D-glucopyranoside.

**Table 2:** Summary statistics of the linear regression analysis of the enzyme uptake

		Coefficient (*β*)				
**Concentration infusion**	**Measurement method**	**Estimate**	**95% CI**	**P value**	Linear regression model (y = α + β * x)	***R^2^***
2 mg/mL	Medium	0.21	(−2.65 to 3.07)	0.86	*y*=131.91 + 0.21 * *x*	0.01
4 mg/mL	Medium	0.35	(−1.49 to 2.18)	0.65	*y*=250.97 + 0.35 * *x*	0.05
2 mg/mL	Fibroblasts	1.61	(−1.09 to 1.89)	0.49	*y*=97.96 + 1.61 * *x*	0.1
4 mg/mL	Fibroblasts	0.55	(−7.37 to 8.48)	0.86	*y*=242.38 + 0.55 * *x*	0.01

Medium: culture medium of the fibroblasts, *R2*: coefficient of determination (proportion of variance explained by the model), *y*: GAA activity measured in the culture medium or cultured fibroblasts (serving as a proxy for cellular uptake of alglucosidase alfa), *α*: estimate of the first measured GAA activity, *β*: slope, *x*: time after preparation of the infusion bags.

A p value for *β* <0.05 is considered statistically significant.

GAA, acid α-glucosidase.

## Discussion

To our knowledge, this is the first study to investigate the stability of alglucosidase alfa in 0.9% sodium chloride solutions for a period longer than 24 hours, as stated in the Summary of Product Characteristic (SmPC), by measuring the GAA activity using 4MU-αGlc and glycogen as substrates and the uptake of GAA enzyme activity in cultured fibroblasts. At both concentrations, 2 and 4 mg/L, no significant change in enzyme activity was observed over 11 days using both substrates. The same applied to the uptake of GAA in cultured fibroblasts, indicating that alglucosidase alfa 2 and 4 mg/L in 0.9% sodium chloride solutions retain functional stability for at least 11 days. These results suggest that prolonged stability may be considered in clinical practice, which would enhance efficiency and flexibility in both infusion preparation and delivery, while concurrently minimising pharmaceutical waste.

It is known that due to the protein-based nature of the product, particle formation may occur in the reconstituted solution and final infusion bags.^12^ In clinical practice, a 0.2 µm in-line filter is used for administration of alglucosidase alfa. Particle formation and particle size distribution over time was not investigated. Aggregated particles may result in less active enzyme available for therapeutic action, diminishing the drug’s efficacy. The initial step of particle formation is often partial or full unfolding of the protein.^23^ When proteins unfold, hydrophobic regions become exposed and tend to clump together as the proteins aggregate to shield these regions from the surrounding water. Moreover, when unfolded, the mannose-6-phosphate (M6P) levels on the surface of the proteins can become misaligned, masked or part of aggregated areas, reducing their ability for receptor binding. Mannose-6-phosphate groups on the enzyme’s surface are key for targeting the enzyme to lysosomes, as they bind to M6P receptors on cell surfaces.^24^ Without proper exposure of M6P, unfolded alglucosidase alfa may have a reduced ability to engage with M6P receptors, decreasing the enzyme’s transport to lysosomes where it would normally act to degrade glycogen. Since the enzymatic activity of alglucosidase alfa is closely related to its efficacy and the enzymatic activity and uptake did not change over the 11 days, there appears to be no apparent loss of protein or activity due to particle formation. Since particle formation is promoted by elevated temperature, further research on the stability of alglucosidase alfa solutions stored at room temperature would be of interest to confirm this assumption.

In this study, the artificial substrate 4MU-αGlc and the natural substrate glycogen were used to quantify alglucosidase alfa enzymatic activity, as this is current standard practice in the diagnosis of Pompe disease in leukocytes at the Center for Lysosomal and Metabolic Diseases of the Erasmus MC.^20^

Cultured human skin fibroblasts are widely used in lysosomal storage disorder research, not only to understand disease mechanisms but also for diagnostic purposes. Therefore, these cells were used for the current enzyme uptake analysis as well. However, a notable disadvantage is the high variability in cultured fibroblasts, as they are sensitive to factors such as cell density and the type of culture medium. This variability in cells could potentially result in deviations in enzyme uptake activity measurement that eventually may render results incomparable. However, this variability was not observed in our analysis. Nonetheless, strict adherence to standardised cell culture protocols remains essential to ensure reliable results for the enzyme uptake and activity analyses.^25^

This study, while offering valuable insights, has certain limitations. Primarily, we did not establish the effects of higher temperatures on the GAA activity. The infusion bags were stored at 2–8°C. It would be of additional benefit to clinical practice if the stability at room temperature (15–25°C) had also been examined, as this information is particularly important in the event that an infusion bag was accidentally stored at room temperature or the cold chain was interrupted on the way to the patient. In this case, the healthcare provider can be supported in his decision whether or not to use the infusion. Second, no investigation into chemical degradation products or structural changes was carried out. While degradation products could influence the stability of alglucosidase alfa infusions and lead to limited efficacy of ERT, they could also trigger an immunological response in patients with Pompe disease.^26 27^ Future studies combining functional assays with analytical testing for degradation, aggregation or other physicochemical changes would be valuable to comprehensively assess the stability profile. Thirdly, we investigated two different concentrations of alglucosidase alfa infusions, 2 and 4 mg/mL, whereas the SmPC of Myozyme® states that the concentration after reconstitution ranges from 0.5 to 4 mg/mL. We deliberately selected this lower concentration limit, as concentrations below this would require administering a large volume, which is not feasible in the home setting or in clinical practice.

## Conclusions

In this study, it was demonstrated that storage of light-protected infusion bags containing 2 and 4 mg/mL alglucosidase alfa at 2–8°C for up to 11 days had no effect on the activity of alglucosidase alfa. Moreover, it did not affect the uptake of alglucosidase alfa in cultured fibroblasts. These findings indicate that both concentrations of the ready-to-administer alglucosidase alfa infusions remain similarly active and provide stable uptake for 11 days compared with the day of preparation. Extending the stability of ready-to-administer alglucosidase alfa infusions could enhance efficiency and flexibility in both infusion bag preparation and home delivery, while concurrently minimising pharmaceutical waste and thus facilitating the introduction of home infusion therapy with alglucosidase alfa worldwide.

## Data Availability

Data are available upon reasonable request.
